# Circular RNA: From non-coding regulators to functional protein encoders

**DOI:** 10.1016/j.pscia.2025.100085

**Published:** 2025-07-30

**Authors:** Xinwei Zhang, Hongyan Wu, Xuechuan Hong, Yuling Xiao, Xiaodong Zeng

**Affiliations:** a School of Pharmacy, Shandong Second Medical University, Weifang, 261053, China; b Shandong Laboratory of Yantai Drug Discovery, Bohai Rim Advanced Research Institute for Drug Discovery Yantai, 264117, China; c State Key Laboratory of Drug Research & Center of Pharmaceutics, Shanghai Institute of Materia Medica, Chinese Academy of Sciences, Shanghai, 201203, China; d Department of Cardiology, Clinic Trial Center, Zhongnan Hospital of Wuhan University, School of Pharmaceutical Sciences, Wuhan University, Wuhan, 430071, China; e Shenzhen Institute of Wuhan University, Shenzhen, 518057, China

**Keywords:** Circular RNA, Structural features, Biological functions, Gene therapy, Delivery strategies

## Abstract

Circular RNAs (circRNAs), a distinct class of non-coding RNAs characterized by a covalently closed circular structure, have gained prominence as a promising research field due to their unique biological properties and functional roles relative to linear RNAs. This review summarizes recent progress in circRNA research, emphasizing fundamental mechanisms and translational potential. We first outline the discovery, biological features, synthesis, and purification of circRNAs. Next, their delivery systems, biological functions, and applications are reviewed. Finally, we discuss challenges and future prospects for clinical translation, with a focus on advancing precision medicine, gene therapy, and personalized vaccines. This review uniquely integrates recent advances in circRNA biology with their translational applications, offering a comprehensive perspective from molecular mechanisms to clinical potential.

## 1 Introduction

Circular RNAs (circRNAs), a distinct class of endogenous non-coding RNAs, have emerged as pivotal regulators in eukaryotic biology due to their covalently closed-loop architecture and multifaceted functional roles. Initially identified in 1976 as circular RNA molecules within the *Solanum tuberosum* spindle tuber viroid [1], circRNAs were later visualized in eukaryotic cytoplasm *via* electron microscopy by Hsu and Coca-Prados in 1979 [2]. Despite these early discoveries, their biological relevance remained obscure until advances in high-throughput sequencing technologies revolutionized the field. A landmark study in 2014 employing systematic RNA sequencing in *Drosophila melanogaster* uncovered over 2500 circRNAs, demonstrating their evolutionary conservation across species [3], including plants [4].

**Fig. 1:**
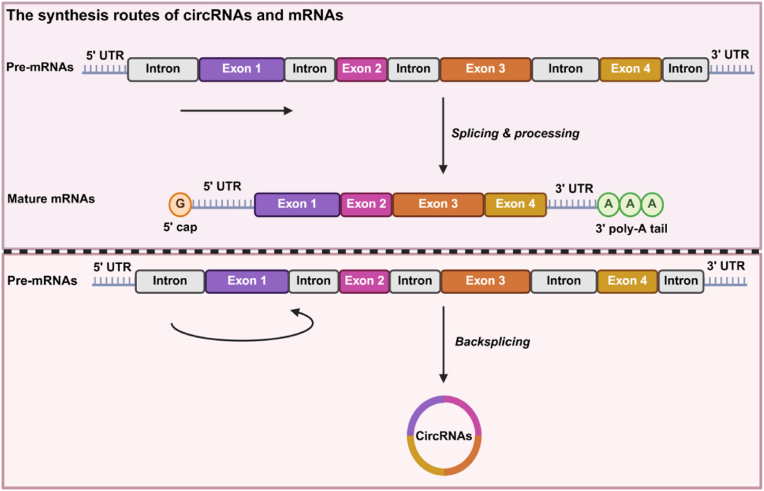
The differences in the synthesis routes of circRNAs and mRNAs. Created in BioRender. zhang, v. (2025) https://BioRender.com/8d1gnmp.

Diverging from linear messenger RNAs (mRNAs), which feature 5′ caps and 3′ poly-A tails, circRNAs are synthesized through back-splicing mechanisms, where downstream 3′ splice sites form covalent bonds with upstream 5′ splice sites [5] (Fig. 1). The closed-loop structure renders circRNAs resistant to exonuclease degradation, conferring exceptional stability and extended half-lives compared to their linear counterparts [6,7]. Such durability underpins their capacity to function as dynamic molecular regulators: circRNAs sequester miRNAs, interact with RNA-binding proteins (RBPs), modulate transcriptional activity, and orchestrate critical pathways implicated in cancer, neurodegenerative disorders (e.g., Parkinson's and Alzheimer's diseases [8,9]), and cardiovascular pathologies [10]. Once dismissed as transcriptional “noise”, circRNAs have undergone a paradigm shift, now recognized as versatile players in gene expression, immune modulation, and other biological processes. Recent breakthroughs further reveal their protein-coding potential, with select circRNAs translated into functional peptides that regulate oncogenic or tumor-suppressive pathways [11].

**Scheme 1:**
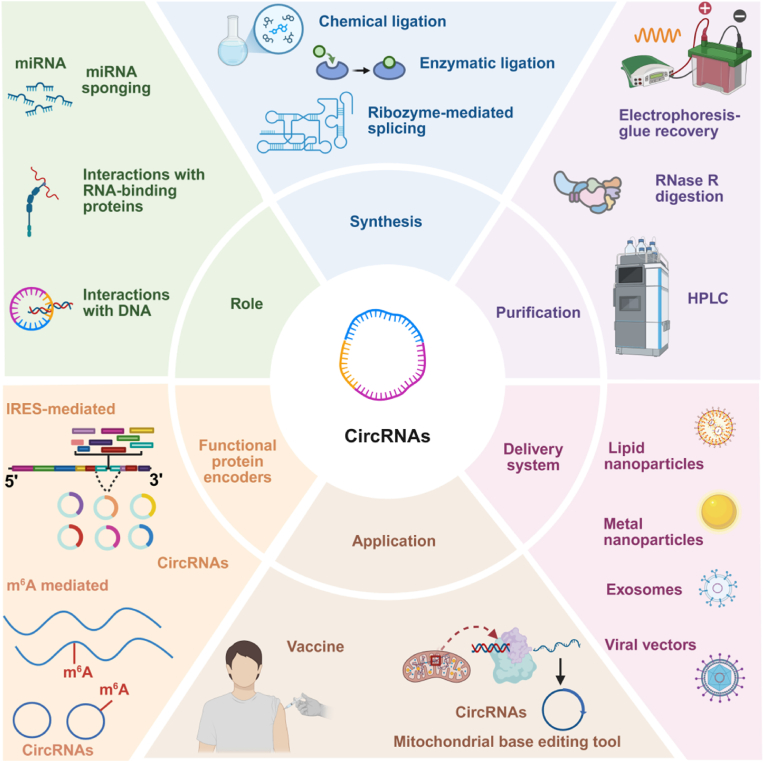
Recent advances in circRNA research. Created in BioRender. zhang, v. (2025) https://BioRender.com/dcy2h8v.

This review (Scheme 1) systematically examines circRNA classification, emphasizing their roles as miRNA sponges, RBP interactors, and transcriptional regulators. We explore their involvement in disease mechanisms across oncology, neurodegeneration, and cardiology, and evaluate emerging applications in synthetic biology. Additionally, we analyze strategies for circRNA synthesis, purification, and delivery, while addressing challenges in clinical translation. Finally, we propose innovative directions to advance circRNA research, with implications for precision medicine, gene therapy, and next-generation therapeutics. In particular, this review highlights the dual role of circRNAs as both non-coding regulators and protein-coding molecules, and underscores their expanding potential in next-generation RNA therapeutics. This unique integrative perspective bridges fundamental mechanisms and translational advances, offering timely insights that distinguish this review within the growing field of circRNA research.

## 2 Classification and functional roles of circRNAs

**Fig. 2:**
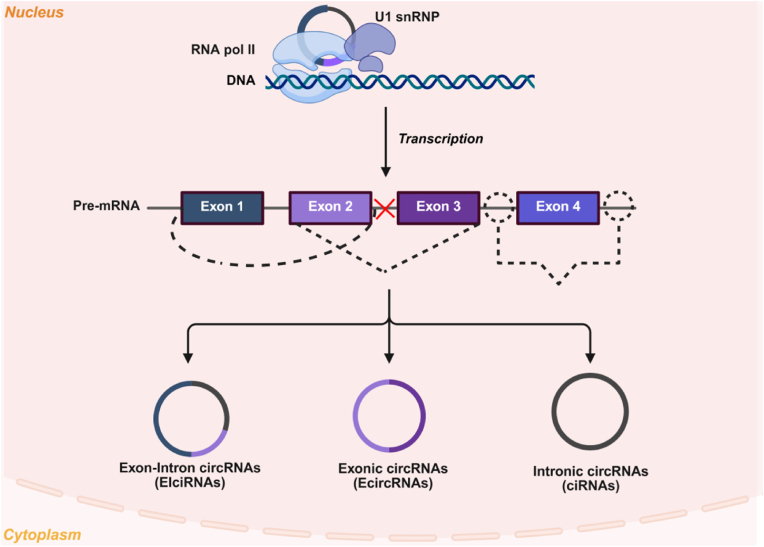
Classification of circRNAs. Created in BioRender. zhang, v. (2025) https://BioRender.com/xcnve4c.

CircRNAs are categorized by genomic origin and splicing mechanism. This process yields three major subtypes: exonic circRNAs, intronic circRNAs, and exon-intron circRNAs (Fig. 2). Exonic circRNAs (EcircRNAs), the most abundant subtype, form *via* back-splicing, where a downstream 3′ splice acceptor ligates to an upstream 5′ splice donor [12]. This circularizes exons, excludes introns, and yields EcircRNAs that translocate to the cytoplasm through nuclear pores [13]. Back-splicing is driven by complementary intronic sequences, such as Alu repeats in humans or reverse complementary motifs in *Drosophila melanogaster*, enabling spliceosome assembly *via* base-pairing [14]. EcircRNAs regulate gene expression by acting as microRNA sponges (e.g., CDR1as/ciRS-7 sequesters miR-7 in neurodegeneration [15]) or binding RBPs like Quaking to stabilize mRNAs [16]. Intronic circRNAs (ciRNAs) arise from lariat introns that evade debranching during pre-mRNA processing [17]. Retained in the nucleus, ciRNAs modulate transcription by interacting with RNA polymerase II (Pol II) at promoters. For example, ci-ankrd52 recruits Pol II to enhance transcriptional elongation noncanonically [18]. Their stability depends on conserved motifs: a 7-nucleotide GU-rich element near the 5′ splice site and an 11-nucleotide C-rich element near the branch point, protecting lariats from degradation [19]. Exon-intron circRNAs (EIciRNAs) retain introns between circularized exons due to partial splicing [20]. Localized to nuclear speckles, they facilitate chromatin interactions *via* RNA-RNA or RNA-protein complexes. For instance, circEIF3J binds U1 small nuclear ribonucleoproteins, forming a complex with Pol II and the EIF3J promoter to boost parental gene transcription [21].

CircRNA functions correlate with subcellular localization [22]. Cytoplasmic EcircRNAs govern post-transcriptional regulation, while nuclear ciRNAs and EIciRNAs influence transcription. This division is conserved evolutionarily; in *Arabidopsis thaliana*, circGORK modulates ion channel expression *via* miRNA sponging [23]. Tools like nanopore sequencing and CRISPR-based screens now enable detailed study of circRNA biogenesis and roles across species, underscoring their biological significance. CircRNAs are stable vaccine vectors, yet *in vivo* degradation and limited cellular uptake due to membrane barriers remain challenges. Adding 2′-O-methyl nucleotides to the 3′ end of PCR templates enhances circRNA resistance to nuclease degradation, extending stability *in vivo* [24]. Furthermore, the structure of circRNAs can trigger immune activation and clearance. Modifying their structure or modulating immune evasion strategies to reduce recognition can enhance their persistence *in vivo*. While these approaches improve stability and delivery efficiency, the mechanisms of circRNAs are intricate, requiring further optimization across different scenarios. Given the biological complexity and therapeutic potential of circRNAs, especially in the context of stability and delivery, it is crucial to further investigate how their structural and sequence features influence not only function but also the efficiency of synthetic approaches—thereby bridging mechanistic insights with practical strategies for circRNA construction and purification.

## 3 Construction and purification of circRNAs

**Fig. 3:**
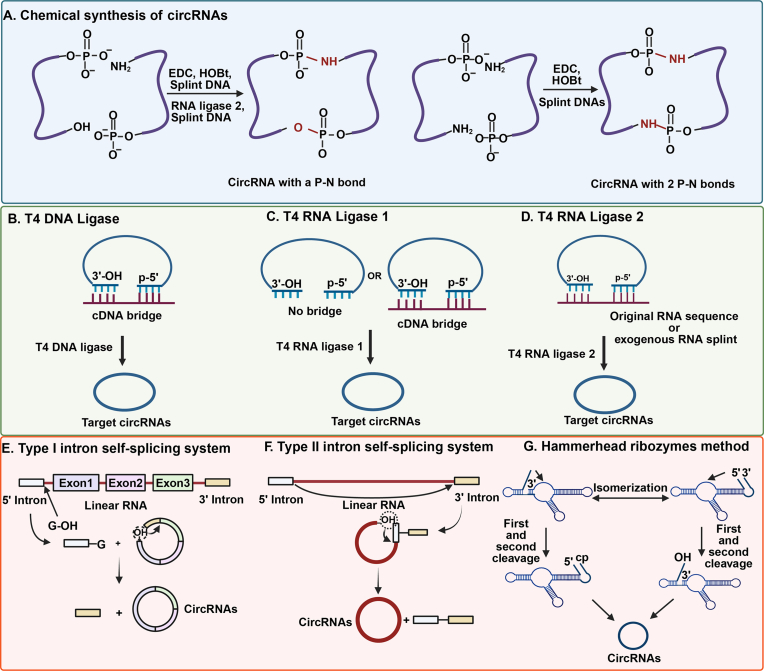
Synthesis of circRNAs. (A) Chemical synthesis of circRNAs. (B) T4 DNA ligase. (C) T4 RNA ligase 1. (D) T4 RNA ligase 2. (E) Type I intron self-splicing mechanism. The type I intron self-splicing mechanism necessitates the inclusion of GTP as a cofactor. (F) Type II intron self-splicing mechanism. (G) Hammerhead ribozymes method. Created in BioRender. zhang, v. (2025) https://BioRender.com/xu679rx.

CircRNAs are generated from linear precursor RNAs that undergo cyclization *via* chemical, enzymatic, or ribozyme-mediated ligation [25]. *In vitro*, three primary methods are employed: chemical ligation, enzymatic ligation, and ribozyme-assisted splicing. Chemical ligation utilizes phosphoramidite chemistry and solid-phase synthesis with nucleotide triphosphate derivatives as starting materials [26] (Fig. 3A). This method forms non-natural ester bonds and suffers from low ligation efficiency, making it less common for circRNA production. Enzymatic ligation, the dominant *in vitro* approach, is divided into T4 ligase-mediated and ribozyme-assisted ligation. Commonly used T4 ligases include T4 DNA ligase (T4Dnl), T4 RNA ligase 1 (T4Rnl1), and T4 RNA ligase 2 (T4Rnl2) (Fig. 3B–D). T4Dnl efficiently ligates double-stranded substrates [27], but its reduced activity with DNA/RNA hybrids limits its utility for RNA ligation compared to double-stranded DNA [28]. T4Rnl1, widely used for RNA circularization, catalyzes the formation of a 5′,3′-phosphodiester bond by joining a 3′-OH group to an activated 5′ end. It prefers specific 5′ and 3′ nucleotide ends and excels at synthesizing circRNAs of 6–8 nucleotides [29]. However, RNA secondary structures and larger sizes decrease its efficiency [30], often causing side reactions like intermolecular ligation, reducing circularization yields. T4Rnl2 is highly effective for RNA ligation, particularly in sealing nicks in double-stranded RNA (dsRNA) rather than single-stranded RNA (ssRNA) ends [31]. Like T4Rnl1, it forms a 5′,3′-phosphodiester bond *via* nucleophilic attack of the 3′-OH on the 5′ end, showing superior efficiency with structured RNAs [29]^.^ RNA scaffolds aid T4Rnl2 in ssRNA ligation, though stability drops for precursors shorter than 30 nucleotides due to spatial constraints [32]. Computational modeling of RNA secondary structures can optimize cleavage sites to enhance ligation efficiency, but outcomes depend on sequence variability [33].

Ribozymes, RNA-based enzymes [34], cyclize precursors *via* three systems: Type I intron, Type II intron, and hammerhead ribozyme synthesis (Fig. 3E–G). The Type I intron self-splicing system, or precursor intron-exon (PIE) method, is the most utilized ribozyme approach. These cis-acting ribozymes self-cleave, followed by exon ligation to form circRNAs [35]. Yu's team developed an enhanced Chimeric Permuted Intron-Exon system utilizing Type I self-splicing introns derived from *Anabaena* pre-tRNALeu and T4 phage thymidylate synthase genes. This platform achieves high-efficiency RNA circularization through dual-module cooperativity, wherein PIE-I facilitates PIE-II folding or circular intermediate generation, significantly minimizing scar sequence retention to reduce potential immunogenicity. Following size exclusion chromatography purification yielding high-purity circRNAs, these circular constructs demonstrated ∼2-fold extended half-lives relative to linear counterparts when expressing EGFP in HEK293 ​cells. In murine models, the engineered circRNA vaccines elicited potent anti-tumor immunity against human papillomavirus (HPV) while simultaneously inducing robust immune activation against SARS-CoV-2 and its variants in COVID-19 vaccine paradigms. Collectively, this work establishes an efficient, versatile cell-free circRNA synthesis platform, providing a robust foundation for diverse circRNA therapeutics including vaccines, protein replacement therapies, and gene editing applications [36]. Compared to chemical and enzymatic methods, PIE offers simpler conditions and purification. Engineered PIE templates enable precise ligation, producing circRNAs without exogenous exons [37], though bacterial-derived sequences may induce immunogenicity [38]. Type II introns produce circRNAs without natural exons by splicing the 5′ site at an exon's 3′ end to the 3′ site at its 5′ end, ensuring accurate precursor ligation [39]. Mali's team developed two RNA circularization methods. One method leverages the *Clostridium tetani* Type II intron for *in vitro* circRNAs' production. In this approach, a linear RNA template is engineered to contain flanking Twister ribozymes. Following transcription, the ribozymes undergo self-cleavage, exposing complementary ligation stems. This triggers the intron's self-splicing reaction, resulting in highly efficient circRNAs' formation. This process achieves a circularization efficiency of approximately 70%, yielding purified circRNAs at 60%. The team validated the expression of these synthetic circRNAs in stem cell-derived neurons and cardiomyocytes, demonstrating significantly more persistent expression compared to linear RNA encoding GFP. Furthermore, they successfully utilized these synthetic circRNAs for targeted genome and epigenome editing *via* fusion with zinc finger proteins and the CRISPR-Cas9 system. This study establishes an efficient, low-immunogenicity platform for circRNAs' production, offering a novel tool for gene therapy, stem cell engineering, and long-lasting gene editing applications [40].

Hammerhead ribozymes, which facilitate *in vivo* genome replication, generate circRNAs through rolling-circle transcription and self-splicing [41]. Linear precursors with hammerhead ribozymes adopt cleavage-active conformations, excising 3′ and 5′ ends to form intermediates with a 5′-hydroxyl and 2′,3′-cyclic phosphate that ligate into circRNAs [32]. *In vitro* transcription using T7 or *Escherichia coli* RNA polymerase yields RNA concatenates that self-cleave and circularize into monomers [34], though the resulting circRNAs are often unstable [42].

In addition, Yu et al. developed a method for direct *in vitro* synthesis of circRNAs using a cis-ligase ribozyme bearing a 5′-triphosphate group. During *in vitro* transcription, the RzL pairs with its substrate sequence and catalyzes the covalent head-to-tail ligation of the linear RNA transcript, resulting in circRNAs' formation. Validation of the synthesized circRNAs revealed that a circRNA driven by a single internal ribosome entry site (IRES) efficiently translated protein. However, inserting two identical IRES elements *in cis* within the same circRNA caused mutual interference, significantly reducing protein expression. Furthermore, circRNAs encoding the natural antiviral protein viperin effectively inhibited infection by JEV-GFP. This study significantly broadens the scope of research into the functional applications of circRNAs for expressing exogenous proteins [43].

Ramakrishnan et al. developed two RNA circularization methods, TRIC and TERIC, based on trans-acting ribozymes. The TRIC method utilizes an *Anabaena* tRNA-derived Type I intron. This intron leverages its internal guide sequence to interact with flanking exons, bringing the P1 and P10 helices into proximity. The target sequence is then attached to the 3′ end of the trans-acting ribozyme, enabling efficient circRNA formation. This method successfully synthesizes circRNAs exceeding 8000 nucleotides in length. The resulting circRNAs exhibit low immunogenicity and sustain prolonged protein expression. Notably, their rolling circle translation efficiency is over 7000-fold higher than that achieved using the OR4F17 IRES. TRIC enables the synthesis of long, modified circRNAs with low immunogenicity, paving the way for clinical therapeutic applications [44].

Each method suits distinct applications: chemical ligation for specific syntheses despite inefficiencies, enzymatic ligation for small or structured RNAs, and ribozyme systems for complex designs. Advances in these techniques improve circRNA synthesis for research and therapeutics, yet challenges in yield, stability, and immunogenicity persist.

**Fig. 4:**
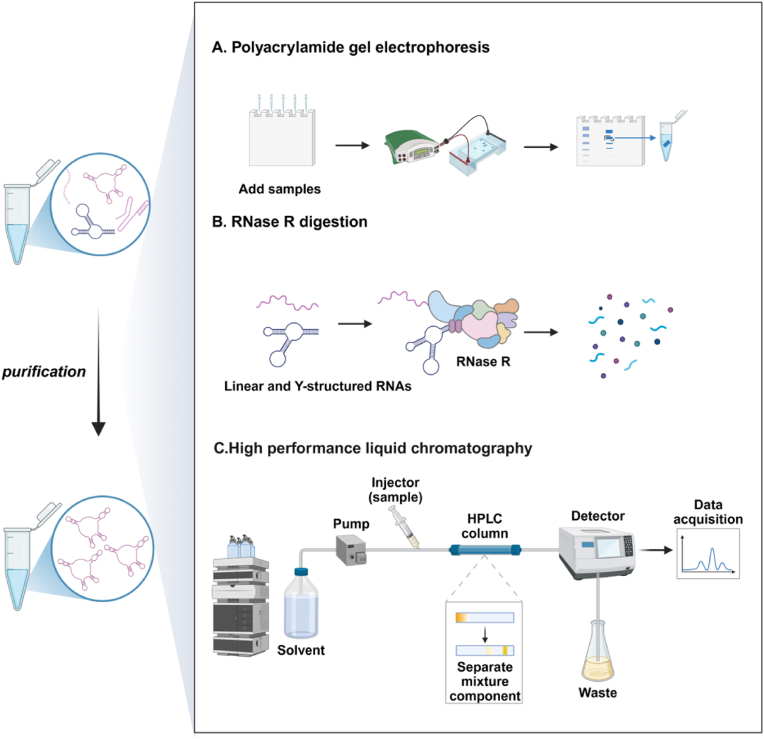
Methods for purification of circRNAs. (A) Polyacrylamide gel electrophoresis. (B) RNase R digestion. (C) High-performance liquid chromatography. Created in BioRender. zhang, v. (2025) https://BioRender.com/lutc6va.

*In vivo* studies of circRNAs require purification to remove impurities and synthesis byproducts. Common methods include polyacrylamide gel electrophoresis (PAGE), RNase R digestion, and high-performance liquid chromatography (HPLC). PAGE separates circRNAs from linear precursors and nicked RNAs using a cross-linked polyacrylamide matrix for molecular sieving and electrophoretic mobility (Fig. 4A). Linear RNAs migrate proportionally to size, while circRNAs, due to their closed-loop structure, migrate slower with aberrant behavior [45]. Though effective for small-scale separation, PAGE is impractical for large-scale use, and heat from gel preparation or electrophoresis may destabilize RNA. RNase R, an exonuclease, degrades linear and Y-structured RNAs, leveraging circRNAs’ resistance to digestion due to their covalent circularity [46] (Fig. 4B). This selectively enriches circRNAs, but excessive digestion can degrade them, requiring optimized conditions for stability and yield [47]. HPLC provides rapid, precise RNA separation with high sensitivity and scalability [48] (Fig. 4C). Its efficiency drops with subtle molecular differences. In 2018, Anderson and colleagues combined size-exclusion chromatography with RNase R digestion, enhancing resolution of circRNAs from linear precursors and nicked RNAs. This yielded circRNAs with >90% purity, supporting both small-scale (gel recovery) and large-scale (HPLC) production, outperforming RNase R digestion alone [49].

Addressing the critical issue of immunogenic impurities (such as dsRNA, 5′-triphosphate groups from un-circularized RNA, and hydrolysis fragments) in synthetic circRNAs triggering innate immune responses and suppressing functionality, the Cao's team developed a multistep purification strategy. This approach combines RNase R digestion to selectively degrade linear RNAs, wood-derived macroporous cellulose chromatography for efficient dsRNA removal, and phosphatase treatment to eliminate 5′-triphosphate groups. This integrated process significantly enhances circRNAs' purity and yield while reducing immunogenicity and mitigating cellular dysfunction [50].

Each method fits distinct needs: PAGE for analysis, RNase R for linear RNA removal, and HPLC for scalable, high-purity output. Challenges in stability and efficiency persist, driving further refinement. For instance, Huang's team has developed a novel cyclization method utilizing the self-splicing capability of the Tetrahymena group I intron ribozyme. This self-targeting splicing (STS) approach achieves simultaneous *in vitro* transcription and cyclization in a single step, overcoming limitations of conventional methods: chemical techniques produce non-natural 2′,5′-phosphodiester bonds with significant side reactions; ligase-based methods require specific substrates and suffer from efficiency constraints; and ribozyme-dependent methods (relying on Type I/II introns) leave immunogenic exon remnants and are limited to RNAs <500 nt. Optimizations to the STS method, including engineering AU-rich sequences into the P1 helix and identifying an optimal antisense sequence length of 150 nt, significantly enhance cyclization efficiency while maintaining stable transcription yields. The resulting circRNAs feature native 3′,5′-phosphodiester linkages without exogenous exon sequences and exhibit low immunogenicity. Following purification by ion-pair reversed-phase HPLC, these circRNAs show minimal innate immune activation. Leveraging this technology, the South Korean company Rznomics is developing circRNAs' therapeutics, with its candidate RXRG001 utilizing this platform having received FDA clearance to enter clinical trials. This STS-based technology provides an efficient, precise, and low-immunogenicity circRNAs' synthesis platform, establishing a foundation for applications in gene therapy, RNA therapeutics, and cancer diagnostic biomarker development [51].While considerable advances have been made in the synthesis and purification of circRNAs, ensuring their efficient and targeted delivery *in vivo* remains a critical challenge.

## 4 The delivery systems of circRNAs

**Fig. 5:**
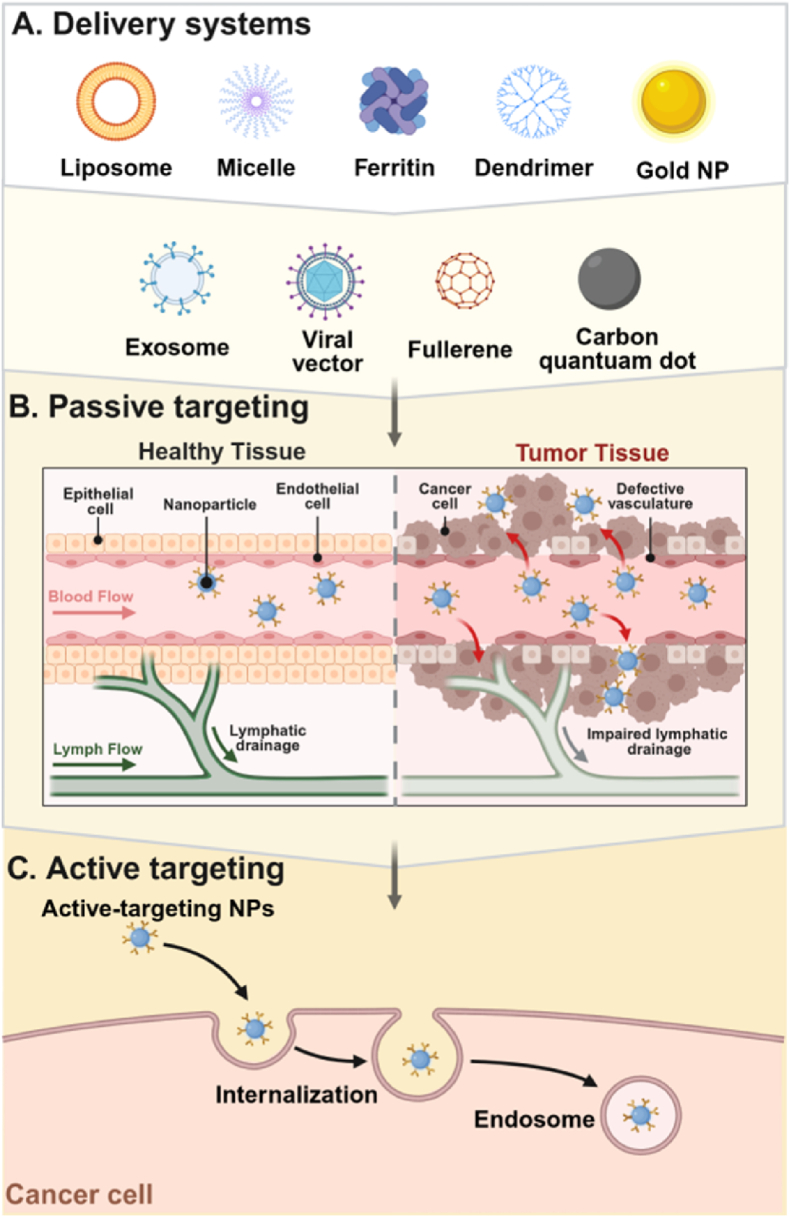
The delivery strategies of circRNAs. (A) Delivery systems. (B) Tumor passive target. (C) Tumor active target. Created in BioRender. zhang, v. (2025) https://BioRender.com/f5cmeat.

In addition to enhancing the stability of circRNAs *in vivo*, an effective delivery system is essential for evading immune system detection. Such technology mitigates degradation risks and facilitates the targeted delivery of circRNAs to their intended sites of expression. Consequently, the meticulous selection and design of delivery vectors are pivotal for the successful application of circRNAs *in vivo* (Fig. 5). Nanoparticle-based carriers are among the most prevalently employed strategies for circRNA delivery (Fig. 5A), significantly improving their stability and cellular uptake.

Liposomal nanoparticles, extensively studied and advanced in clinical settings, are composed of cationic or ionizable lipids, cholesterol, helper lipids, and polyethylene glycol (PEG) [52]. These nanoparticles encapsulate circRNAs within their bilayer membranes, safeguarding them from *in vivo* degradation and enabling both delivery and targeted transport functions [53]. For instance, high-throughput combinatorial approaches have been utilized to synthesize and screen lipid nanoparticles specifically targeting the lungs. One such ionizable lipid nanoparticle, tailored for tumor targeting, has been shown to deliver circRNAs to the lungs, thereby enhancing immunotherapy for lung cancer [54]. Despite their favorable biocompatibility, liposomes exhibit relatively limited delivery efficiency and drug-loading capacity, necessitating further optimization.

Zhao's team developed a circRNAs' vaccine encoding both an antigen (e.g., influenza HA or SARS-CoV-2 RBD) and the chemokine CXCL13 to enable co-expression. Co-delivery *via* lymph node-targeting lipid nanoparticles (LNPs) enhanced cross-reactive antibody responses and protected mice against both homologous and heterologous influenza virus challenges. Antibody-modified LNPs also improved the stability of the lyophilized vaccine. This approach offers a promising strategy for broad-spectrum vaccine development [55].

Building on their work with synthetic compounds that form complexes with RNA for delivery, Zhang et al. have developed a novel carrier, Guanidinium Serine-Cleavable Amphiphilic RNA Transporters (GSer-CARTs), evolving from traditional guanidinium groups. The RNA complex exhibits stability in acidic formulations but undergoes rapid degradation at physiological pH, enabling efficient payload release. Researchers successfully utilized GSer-CARTs to deliver circular RNAs encoding ovalbumin (circOVA), which elicited a potent antigen-specific CD8^+^ T cell response and significantly suppressed melanoma tumor growth in a murine model. This successful application of circOVA delivery in cancer vaccination underscores the translational promise and clinical potential of the GSer-CART platform, highlighting its broader utility for diverse biomedical research and therapeutic applications [56].

Metal nanoparticles, such as gold nanoparticles, recognized for their robust optical properties and surface modification capabilities, represent another promising avenue for circRNA delivery. CircDnmt1 has been identified as being overexpressed in breast cancer, facilitating the nuclear translocation of p53 and AUF1, thereby stimulating tumor growth and autophagy pathways. Investigations have employed PEG-modified gold nanoparticles as delivery systems for siRNA aimed at circDnmt1. In a murine model of breast cancer, a significant reduction in tumor volume was observed [57]. CircAmotl1 plays a significant role in enhancing the process of wound healing. In a murine model of skin injury, the application of gold nanoparticles that deliver circAmotl1 expression plasmids promotes the translocation of STAT3 into the cell nucleus, thereby markedly expediting the healing of skin wounds [58].

While these nanoparticles offer advantages in stability and delivery efficiency, potential cytotoxicity and biosafety concerns must be addressed [59]. Viral vectors, leveraging the natural infection mechanisms of viruses, enable efficient delivery of circRNAs into cells. For example, virus-like nanoparticles derived from HPV have been constructed to encapsulate circRNAs, overcoming challenges associated with cellular uptake and membrane penetration. Specifically, HPV-lipid nanoparticles, combined with the transmembrane peptide L17E, enhance stability and targeting, promoting endosomal release and providing an innovative approach for circRNA delivery [60].

Exosomes, with their exceptional properties—including high stability, low immunogenicity, and the ability to penetrate cellular barriers—serve as ideal vehicles for circRNA delivery. These extracellular vesicles, first identified in the reticular cells of sheep, are characterized by their lipid bilayer structure and a size range of 50–100 nm [61]. For therapeutic applications, circRNA mSCAR has been encapsulated in exosomes and successfully delivered *via* TPP-PDL electroporation to intervene in sepsis and other inflammatory diseases [62]. Exosomes exhibit unique biocompatibility and pharmacokinetic properties; however, challenges in their scalable manufacturing remain a critical hurdle.

Furthermore, the investigation of carbon-based nanoparticles and ferritin as delivery systems has experienced substantial growth over the past decade, garnering considerable interest across various domains, including drug delivery, gene therapy, imaging, and biosensing. While applications focused on circRNAs remain in the early stages of development, the body of research concerning carbon-based nanomaterials as carriers for nucleic acid delivery is extensive and holds significant theoretical and practical implications. The mechanisms by which nanoparticles target drug or nucleic acid delivery can be categorized into two primary types: passive targeting and active targeting (Fig. 5B and C). Both strategies are fundamental to the design of nanocarrier delivery systems and are particularly critical in the context of tumors, cardiovascular diseases, inflammatory conditions, and gene therapy [63].

Enhancing the specificity of circRNA delivery is a pivotal step in maximizing its therapeutic efficacy. Targeted delivery not only mitigates off-target effects but also significantly improves therapeutic outcomes. To achieve precise targeting, specific ligands, such as peptides [64], can be conjugated to the surface of delivery carriers, enabling the selective recognition of target cells or tissues. For example, receptors uniquely expressed on the surface of tumor cells can serve as targets, thereby improving the selective delivery of circRNAs to tumor cells. The physicochemical properties of delivery carriers, including particle size, surface charge, and hydrophilicity, can be modulated to enhance their affinity for specific cell types, further optimizing targeted delivery efficiency. In the context of brain delivery, overcoming the blood-brain barrier (BBB) remains a major challenge. Several strategies have been proposed to address this limitation. For instance, ligands capable of binding to transport receptors within the BBB, such as transferrin [65]and lactoferrin [66], can be attached to delivery carriers to facilitate the transcytosis of circRNAs across the barrier. In addition to ligand-based approaches, physical methods such as ultrasound, pulsed electric fields, or drug-induced techniques have been employed to transiently disrupt the BBB [67]. These methods enhance the permeability of the barrier, enabling more efficient delivery of circRNAs to the brain. Building upon advances in delivery technologies, circRNAs can now be efficiently and selectively transported into specific tissues and cells, allowing them to exert their biological functions and paving the way for their application in disease treatment.

## 5 Biological function and application of circRNAs

### 5.1 miRNA sponging

The concept that circRNAs can function as “sponges” for miRNAs was first introduced in 2013. Subsequent empirical studies have established that circRNAs act as competitive endogenous RNAs, often harboring multiple miRNA-binding sites. Through these interactions, circRNAs bind and sequester miRNAs, preventing them from interacting with their target mRNAs and thereby attenuating the repressive effects of miRNAs on gene expression [68]. Given that miRNAs are small, evolutionarily conserved molecules that regulate approximately 30% of protein-coding genes [69], circRNAs play a critical role in modulating transcriptional regulatory networks. Applications of circRNA sponges have been explored in various disease contexts. For instance, synthetic circRNA sponges have been developed as miRNA inhibitors to mitigate cardiac hypertrophy induced by pressure overload [70]. Additionally, circCACTIN has been identified as a sponge for miR-331–3p, modulating TGFBR1 expression and promoting gastric cancer progression [71].

Among circRNAs, CDR1as (also known as ciRS-7) stands out as a prominent miRNA sponge. It contains a high number of miR-7-binding sites, with 130 sites in murine models and 73 sites in humans [72]. CDR1as plays a significant role in regulating gene expression and developmental processes, particularly within the nervous system. Recent studies have demonstrated that following spinal cord injury, circRNA CDR1as functions as a miRNA sponge by inhibiting miR-7a-5p activity. This inhibition directly enhances the expression of TGF-βR2, promoting fibrosis and impairing nerve regeneration [73].

**Fig. 6:**
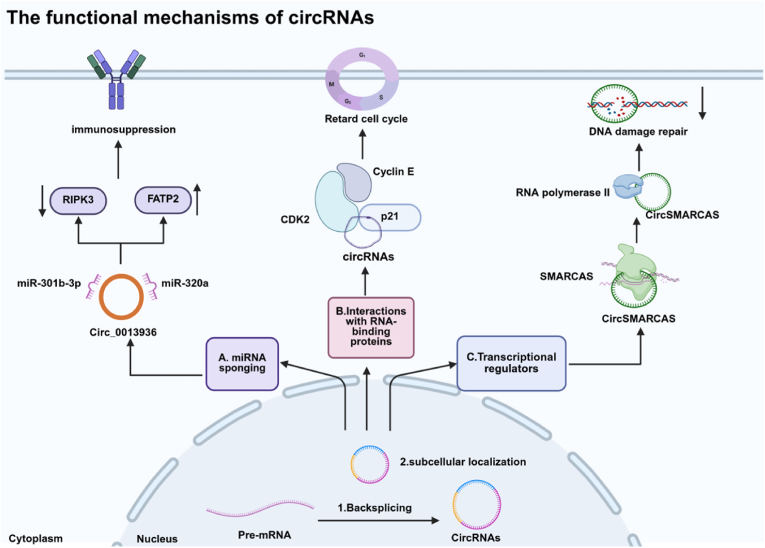
The functional mechanisms of circRNAs. (A) miRNA sponging. (B) Interactions with RNA-binding proteins. (C) Transcriptional regulators. Created in BioRender. zhang, v. (2025) https://BioRender.com/jge0iki.

Bladder cancer, a common urinary system malignancy with poor prognosis in invasive cases, involves polymorphonuclear myeloid-derived suppressor cells (PMN-MDSCs)—pathologically activated immune cells exhibiting strong immunosuppression. Tan's team revealed that tumor-derived exosomes deliver circRNA_0013936, which is phagocytosed by PMN-MDSCs. Functioning as a miRNA sponge, circRNA_0013936 sequesters miR-320a and miR-301b-3p, leading to the upregulation of FATP2 (a lipid metabolism molecule promoting immunosuppressive molecule synthesis) and the downregulation of RIPK3 (enhancing immunosuppressive function), respectively. These dual pathways synergistically amplify PMN-MDSC-mediated immunosuppression. Consequently, circRNA_0013936 represents a promising novel immunotherapy target for bladder cancer, as blocking its function may restore anti-tumor immune responses [74](Fig. 6A).

### 5.2 Interactions with RNA-binding proteins

Numerous circRNAs are predicted to interact with RBPs [75], playing critical roles in a wide range of cellular physiological functions. These interactions occur primarily through two mechanisms: direct binding and scaffold-mediated complex formation. In the case of direct binding, circRNAs interact with RBPs *via* specific sequence and structural motifs that exhibit complementarity. The spatial conformations of both molecules further influence the binding affinity between circRNAs and RBPs. For example, during the cell cycle, cyclin E binds to CDK2 to facilitate gene expression and promote the transition from the G1 phase to the S phase [76]. The cyclin-dependent kinase inhibitor p21, on the other hand, binds to CDK2 and inhibits its activity [77]. Circ-Foxo3 disrupts this process by simultaneously interacting with both p21 and CDK2, thereby preventing CDK2 from binding to cyclin E. This interaction results in a slowdown of cell cycle progression [78](Fig. 6B).

CircRNAs can also directly interact with chromatin-interacting RNA-binding proteins (ChRBPs), which play crucial regulatory roles in gene transcription. Huang's team identified the ChRBP gawky as a specific regulator in stress-induced transcription (Gawky modulates MTF-1-mediated transcription activation and metal discrimination). Under copper stress, they identified a class of circRNAs containing metal-responsive element (MRE). These MRE circRNAs bind to ChRBP gawky and modulate its function. Overexpression of MRE circRNAs significantly inhibited the transcriptional activation of stress-response genes, exacerbated copper-induced DNA damage, reduced cellular resistance to copper stress, and facilitated the clearance of damaged cells [79].

In addition to direct binding, circRNAs can also act as scaffolds to facilitate the assembly of functional complexes involving multiple RBPs. These complexes perform diverse cellular functions. For instance, circCLASP2 serves as a molecular connector, mediating the interaction between the RNA helicase DHX9 and PCMT1 mRNA [80]. This interaction enhances ribosomal enrichment, promotes cytoskeletal reorganization, and facilitates tumor metastasis [81]. Furthermore, circRNAs can influence the subcellular localization of RBPs. For example, circHIF1A interacts with the NFIB protein, promoting its translocation to the nucleus. This nuclear localization activates the downstream AKT/STAT3 signaling pathway and inhibits the expression of p21 [82].

### 5.3 The role of circRNAs as transcriptional regulators

CircRNAs can influence the transcription of associated genes by forming complexes with RNA Pol II [83]. These interactions serve as a mechanism for circRNAs to regulate Pol II transcriptional activity positively. For example, EIciRNAs and circAIP2 interact with U1 snRNPs and Pol II to modulate the transcription of their host genes, thereby enhancing transcriptional output [84]. Another example is circ-DONSON, which is highly expressed in gastric cancer tissues. circ-DONSON facilitates the transcriptional activation of SOX4 by interacting with the nucleosome remodeling factor complex at the SOX4 promoter region, contributing to gastric cancer progression [85].

In addition to their interactions with RNA and proteins, circRNAs can directly interact with DNA through complementary sequence pairing, forming circular RNA-DNA complexes, commonly referred to as circR-loops. These complexes play a pivotal role in regulating gene expression. When circR-loops form in promoter or enhancer regions, they can enhance transcriptional activity. Conversely, when located within gene bodies, they may influence alternative splicing by modulating the selection of splice sites. However, the formation of circR-loops can also have detrimental effects. These structures have the potential to induce double-strand breaks in DNA, which interfere with normal DNA repair mechanisms. This disruption can result in genomic instability and the accumulation of mutations, further contributing to disease progression [86].

CircSMARCAS, generated through back-splicing of exons 15–16 of the SMARCA5 gene, is significantly downregulated in breast cancer tissues and cell lines, while its host gene SMARCA5 exhibits elevated expression. CircSMARCAS binds to the exon 15 region of the SMARCA5 genomic locus, forming a R-loop that induces RNA polymerase II stalling during exon 15 transcription. This transcriptional arrest promotes premature termination, generating nonfunctional truncated SMARCA5 proteins while reducing full-length SMARCA5 expression. Given SMARCA5's critical role in DNA damage repair, circSMARCAS overexpression compromises cellular DNA damage repair capacity. Paradoxically, xenograft models overexpressing circSMARCAS demonstrate significantly enhanced response to cisplatin therapy. Furthermore, circSMARCAS sensitizes breast cancer cells to both cisplatin and bleomycin. Consequently, restoring circSMARCAS expression represents a promising strategy to reverse chemoresistance and provides a novel therapeutic approach for breast cancer [87](Fig. 6C).

### 5.4 CircRNAs as functional protein encoders

In 2017, the first two protein-coding circRNAs were identified: circZNF609 [88], discovered in myogenic cells, and circMbl [89], identified in synaptosomes. Since then, increasing evidence has demonstrated that circRNAs can be translated into peptides and proteins, thereby playing critical roles in modulating diverse physiological processes. Unlike traditional mRNAs, which feature a 5′ cap and a 3′ poly-A tail, circRNAs lack these canonical structures, necessitating distinct translation mechanisms.

**Fig. 7:**
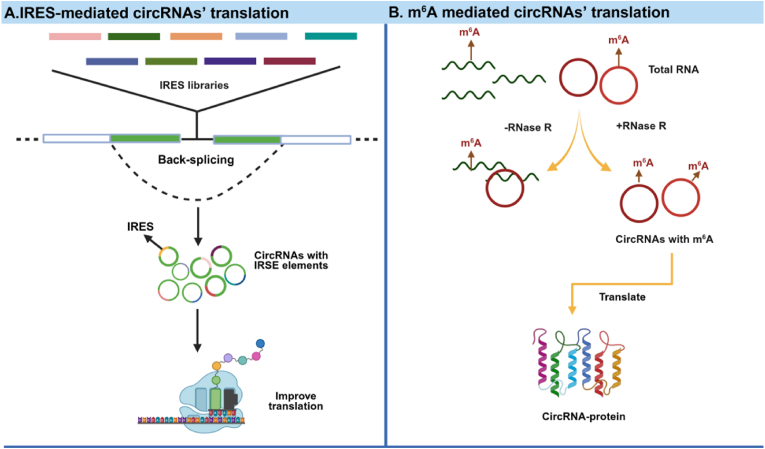
Schematic diagrams of IRES (A) and m^6^A (B) mediated circRNA translation. Created in BioRender. zhang, v. (2025) https://BioRender.com/vc8zkdt.

The protein-coding potential of circRNAs is primarily mediated through cap-independent translation pathways. These include IRES-dependent translation and N^6^-methyladenosine (m^6^A) modification-mediated translation, both of which enable efficient translation in the absence of a standard 5′ cap structure (Fig. 7). These unique translation mechanisms highlight the regulatory complexity and functional versatility of circRNAs in cellular physiology.

IRES is an RNA sequence, typically 150 to 250 base pairs in length, that facilitates the binding of the 40S ribosomal subunit upstream of the translation initiation codon, thereby enabling cap-independent initiation of protein translation [90]. The discovery of IRES sequences in circRNAs dates back to 1995, when studies revealed their presence upstream of translation initiation codons, suggesting that IRES elements and open reading frames (ORFs) could drive cap-independent translation [91]. Building on these findings, a high-throughput screening method was developed in 2021 to systematically identify RNA sequences capable of guiding circRNA translation in human cells. This approach identified over 17,000 natural and synthetic sequences as potential IRES elements for circRNAs. Among these findings, the protein-coding circFGFR1p was identified as being downregulated in cancerous conditions. circFGFR1p functions as a negative regulator of the FGFR1 oncoprotein, inhibiting cellular proliferation under stress conditions [92].

In addition, circ-EIF6 was found to promote both proliferation and metastasis of triple-negative breast cancer cells *in vitro* and *in vivo*. Yang et al. identified a 675-nucleotide ORF within circ-EIF6 that encodes a novel 224-amino acid peptide, EIF6-224. This peptide is implicated in the oncogenic properties of circ-EIF6 and directly interacts with the oncogene MYH9 in breast cancer. These findings suggest a potential therapeutic strategy for targeting triple-negative breast cancer [93]. Collectively, these studies underscore the critical role of IRES elements in promoting circRNA translation and their relevance in cancer biology [94].

m^6^A modification, the most prevalent RNA modification in eukaryotic organisms, plays a pivotal role in regulating RNA splicing, export, translation, and stability [95,96]. This modification is catalyzed by m^6^A methyltransferases and removed by demethylases, with its recognition mediated by m^6^A-binding proteins [97]. Research has shown that circRNAs often contain consensus m^6^A motifs, and even a single m^6^A site is sufficient to initiate translation. Further studies have demonstrated that m^6^A-mediated translation of circRNAs is widespread across various biological contexts [98]. For example, circE7 undergoes m^6^A modification and is translated into the E7 oncoprotein, which drives the proliferation of HPV-infected cells [99]. In male germ cells, approximately 50% of circRNAs contain significant ORFs with m^6^A-modified initiation codons at their junctions. Using liquid chromatography-mass spectrometry, researchers have detected hundreds of peptides encoded by circRNA junction sequences, indicating that circRNAs can be translated into proteins in both immature and mature male germ cells [100].

Both IRES and m^6^A modifications constitute key mechanisms driving circRNAs' translation. Their relative efficiency and predominance are co-determined by circRNA-specific sequence features, the molecular milieu of host cells, and specific physiological or pathological stimuli—no single mechanism can be categorically deemed universally predominant. CircRNAs lack traditional translation initiation elements, so they rely on the IRES mechanism to initiate translation [101]. IRES represents a canonical cap-independent translation mechanism that predominates under robust stress stimuli where cap-dependent translation is suppressed. m6A is the most common internal modification in mammalian mRNA, influencing processes such as mRNA transcription, pre-mRNA splicing, nuclear export, mRNA stability, and translation. The m^6^A modification of circRNAs may also undergo internal translation initiation through similar mechanisms [98]. In pathological contexts characterized by dysregulation of m^6^A-related enzymes (e.g., cancer), the m^6^A pathway frequently assumes a critical and pervasive role in circRNAs’ translation.

### 5.5 CircRNA markers associated with various cancers

**Fig. 8:**
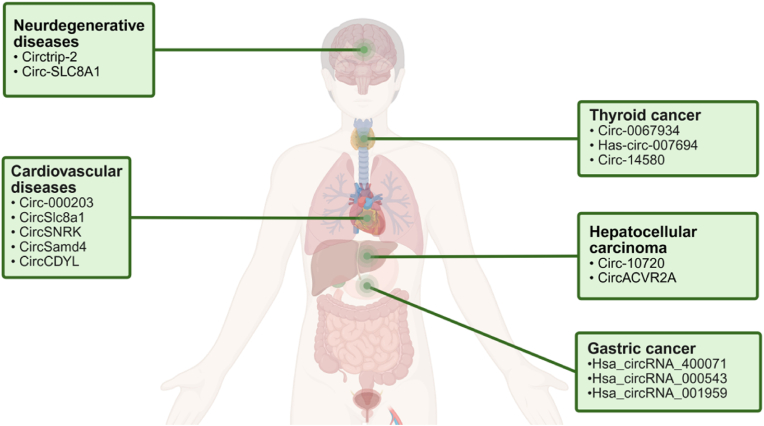
CircRNA markers associated with various diseases. Created in BioRender. zhang, v. (2025) https://BioRender.com/nfybz7b.

Tumor cell metastasis is a leading cause of cancer-related mortality, and emerging evidence highlights the critical role of circRNAs in this process (Fig. 8). circRNAs have been shown to enhance tumor cell migration and invasion by modulating miRNA activity or directly interacting with cytoskeleton-associated proteins. For instance, in laryngeal squamous cell carcinoma, circRNAs bind to miR-1207–5p, potentially driving oncogenic progression and facilitating tumor cell metastasis [102].

The acceleration of modern lifestyles has contributed to a rising prevalence of diseases among younger populations, including thyroid-related disorders. Notably, the incidence of thyroid cancer has steadily increased over time, making it the third most common cancer in terms of national prevalence [103]. Among thyroid cancer subtypes, papillary thyroid carcinoma (PTC) is the most frequently diagnosed. Recent research has identified numerous circRNAs associated with thyroid cancer, shedding light on their potential roles in disease progression [104]. For instance, circ_0067934 functions as a sponge for miR-1304, thereby modulating the expression of CXCR1. This regulatory mechanism suppresses cell proliferation, migration, and invasion while promoting apoptosis in thyroid cancer cells [105]. Similarly, hsa_circ_0007694 is downregulated in PTC cells and exhibits tumor-suppressive effects, including enhanced apoptosis, reduced cancer cell proliferation, migration, and invasion, and inhibition of tumor growth [106]. Additionally, circ_14580 directly binds to cathepsin W, regulating fibroblast activation and fibrosis in PTC. This interaction provides new insights into the functional roles of circRNAs in PTC progression and highlights their potential as therapeutic targets [107].

Gastric cancer remains an aggressive malignancy and, despite advancements in nutrition, food preservation, and therapeutic strategies, it continues to rank as the fourth most prevalent cancer globally, albeit with a declining incidence rate [108]. Recent studies employing Agilent microarray technology, followed by validation via qRT-PCR, have identified significant alterations in the expression profiles of circRNAs and mRNAs. Among these, 1285 circRNAs were found to exhibit differential expression and possess corresponding miRNA binding sites, such as hsa_circRNA_400071, hsa_circRNA_000543 and hsa_circRNA_001959. These circRNAs regulate gene expression by interacting with miRNAs, highlighting their potential as novel molecular biomarkers for gastric cancer [109].

Building on these findings, recent research has introduced a novel dual-modal detection platform, BEISA, which leverages extracellular vesicle-derived circRNAs from gastric cancer cells. This platform integrates fluorescence and chemical signal detection of extracellular vesicle-derived circRNAs, with data analysis performed using machine learning algorithms. The integration of these modalities significantly enhances the accuracy of gastric cancer diagnosis and improves early detection capabilities [110].

Hepatocellular carcinoma (HCC) is one of the most prevalent cancers and a leading cause of cancer-related mortality worldwide. In 2020, approximately 906, 000 new cases of liver cancer were diagnosed globally, with a five-year relative survival rate for HCC of approximately 18% [111,112]. Twist1, a key transcription factor that promotes epithelial-mesenchymal transition (EMT), regulates the expression of EMT-associated genes by activating or inhibiting their promoters. Twist1 increases the levels of mesenchymal markers, such as vimentin, while simultaneously reducing the expression of epithelial markers, such as E-cadherin [113]. Recent studies have demonstrated that Twist1 modulates the expression of Cullin2 circRNAs, which enhance vimentin expression by interacting with its promoter. Specifically, Twist1 selectively increases the transcription of circ-10720. In HCC, circ-10720 levels are positively correlated with Twist1 expression and tumor aggressiveness, as circ-10720 sequesters miRNAs targeting vimentin. By upregulating circ-10720, Twist1 facilitates vimentin expression, providing potential therapeutic targets and new avenues for HCC treatment [114]. In addition to circ-10720, circACVR2A has been identified as a critical regulator in HCC. circACVR2A is significantly upregulated in HCC cells and interacts with miR-511-5p to activate the PI3K-Akt signaling pathway. This activation drives the upregulation of key oncogenes, including PIK3R3, ERBB4, and KRAS, thereby suppressing apoptosis and promoting HCC cell proliferation, migration, and tumor progression. These findings highlight circACVR2A as a promising therapeutic target for HCC treatment [115].

### 5.6 CircRNA markers associated with neurodegenerative diseases

Neurodegenerative diseases encompass a group of neurological disorders characterized by the progressive and relentless degeneration of specific neurons and axons within the central nervous system [116]. Among these, Alzheimer's disease (AD) and Parkinson's disease (PD) are among the most prevalent conditions [117]. AD is an age-associated neurodegenerative disorder marked by a gradual decline in cognitive function [118]. Diagnosis of AD is typically based on the presence of β-amyloid plaques and hyperphosphorylated tau proteins [119]. The association between neurodegeneration and the MAPT gene was first identified in FTLDMAPT, a condition caused by several mutations at the MAPT locus on chromosome 17 [120]. Notably, the MAPT locus also generates circRNAs, suggesting a potential role for circRNAs in the pathogenesis of neurodegenerative diseases ^[^ [121].

PD is another neurodegenerative disorder linked to brain aging, clinically characterized by bradykinesia, rigidity, and tremors [122]. In transgenic *Caenorhabditis elegans* models of PD, a novel circRNA, circzip-2, was discovered. Reducing circzip-2 levels decreased α-synuclein aggregation, a hallmark of PD pathology, and extended the lifespan of the worms ^[^ [123]. Bioinformatics analyses have revealed that differentially expressed circRNAs in PD models are involved in various biological processes, including synaptic function, disease development and progression, axonal guidance, and signaling pathways [124]. Among these, circ-SLC8A1 (Fig. 8) was found to be upregulated in the dopaminergic regions of the brains of PD patients and in oxidative stress cell models [125]. The rapid advancement of high-throughput sequencing technologies has identified numerous circRNAs associated with neurodegenerative diseases. However, their specific functional roles remain largely unknown. Elucidating the functions of these circRNAs could deepen our understanding of aging processes and provide novel therapeutic opportunities for treating neurodegenerative diseases [126].

### 5.7 CircRNA markers associated with cardiovascular diseases

CircRNAs have emerged as key players in cardiovascular diseases (Fig. 8), with accumulating evidence highlighting their involvement in processes such as mitochondrial dynamics and apoptosis [127]. Advances in deep sequencing technologies and refined data analysis techniques have further elucidated the critical roles of circRNAs in various cardiovascular pathologies, including myocardial infarction, cardiac fibrosis, and primary cardiomyopathies. In hypertrophic cardiomyopathy, circUtrn stabilizes the protein pp5, activating the Erk1/2 signaling pathway and promoting cardiac hypertrophy. Similarly, circRNA_000203 and circSlc8a1 act as miRNA sponges, enhancing hypertrophic responses. In the context of myocardial infarction, circNfix promotes degradation of Ybx1, inhibiting cell proliferation and angiogenesis, while circSNRK sponges miR-33 to enhance energy production. Additionally, circSamd4 interacts with Vcp to increase oxidative stress. In diabetic cardiomyopathy, circRNA DICAR mediates the degradation of Med12 to suppress pyroptosis, whereas circRNA CACR and circHIPK3 promote pyroptosis and fibrosis by targeting miR-214–3p and miR-29b-3p, respectively. In doxorubicin-induced cardiotoxicity, circFoxo3 prevents the nuclear translocation of key factors to inhibit cellular senescence, while circ-Amotl1 enhances cardiomyocyte proliferation *via* the AKT1 pathway. circNlgn translates Nlgn173, which promotes apoptosis and fibrosis, whereas circ-INSR stabilizes mitochondrial DNA through SSBP1, thereby preventing apoptosis and maintaining mitochondrial homeostasis^[^ [128].

Specifically, circNCX1 sequesters miR-133–3p, upregulating the expression of the pro-apoptotic protein CDIP1 and modulating myocardial cell apoptosis [129]. Additionally, circCDYL promotes cardiomyocyte proliferation *via* the miR-4793-5p/APP signaling pathway [130]. Comprehensive RNA sequencing of ribosomal RNA-depleted samples from 12 human hearts, 25 mouse hearts, and cardiomyocytes derived from 28-day-old human embryonic stem cells has revealed a robust circRNA expression profile. This study uncovered an abundance of heart-specific circRNAs, paving the way for future investigations into their roles in cardiac pathophysiology [131].

### 5.8 Application of circRNAs

**Fig. 9:**
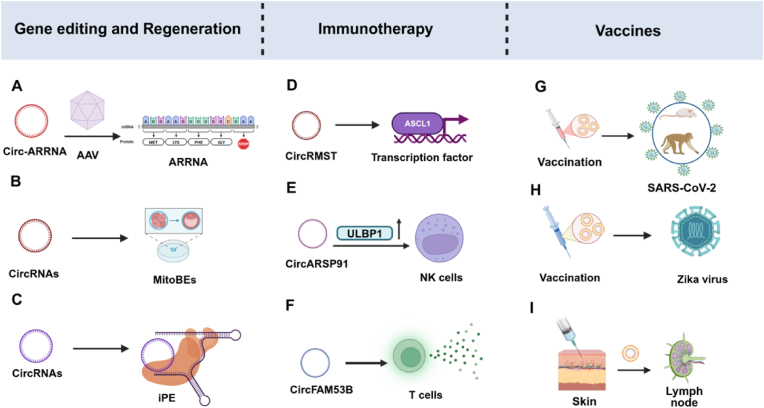
Application of circRNAs. Created in BioRender. zhang, v. (2025) https://BioRender.com/irllph9.

Advances in RNA biology and gene editing technologies have underscored the significant potential of circRNAs across diverse fields. In 2022, Wei et al. designed and utilized a circular RNA (circ-ARRNA) that can recruit ADAR. Through adeno-associated virus (AAV) delivery, the genetically encoded circ-ARRNA was able to achieve long-term RNA editing in human primary cells and organoids. This circular RNA, which avoids cleavage by exonucleases, further enhanced the efficiency and accuracy of editing both *in vitro* and *in vivo*, and essentially eliminated off-target editing on the target transcripts within the double-stranded RNA region [132] (Fig. 9A). In 2023, they further utilized AAV to deliver the engineered circ-ARRNA into non-human primates and humanized mice, achieving long-term, efficient and precise RNA base editing [133]. Subsequently, they introduced an innovative mitochondrial base editing tool (Fig. 9B), the mitoBEs system, which integrates TALE proteins, nicking enzymes, and deaminases to enable precise and efficient editing of mitochondrial DNA. By encoding the mitoBEs system in circRNAs, successful editing was achieved in cellular models such as HEK293T and H1299, overcoming challenges associated with CRISPR technology in mitochondrial contexts and enhancing delivery efficiency [134]. Further refinements, including optimized deaminase activity and cytidine deaminase substitution, enabled the generation of mouse models with Leigh syndrome and Leber's hereditary optic neuropathy phenotypes. These models exhibited stable inheritance of edited mutations for up to six months postnatally, establishing a high-precision platform for mitochondrial disease research and therapeutic development [135].

Conventional prime editors rely on the nCas9-H840A nickase, enabling precise edits only downstream of the cleavage site and lacking an efficient reverse editor (iPE) for upstream editing. Recently, Gao's team developed a circRNA-mediated iPE system by replacing nCas9-H840A with nCas9-D10A, achieving upstream editing. This system utilizes circRNA to deliver both the reverse transcription template and the primer binding sit, forming an R-loop structure that facilitates DNA unwinding. The team further enhanced DNA unwinding capability by incorporating the *E. coli*-derived 3′ to 5′ helicase Rep-X, boosting editing efficiencies to 2.7%–55.4%. In breast cancer models, BRCA1 editing efficiency reached 13.3%, while in Leber congenital amaurosis models, RPE65 editing efficiency was 9.5%. By combining nCas9-H840A and nCas9-D10A-based systems, this iPE platform achieves genome-wide coverage, providing a pioneering approach for correcting pathogenic mutations located upstream of the cleavage site [136] (Fig. 9C).

In cardiovascular research, circRNA-RBCK1 has been shown to mitigate heart failure by binding to miR-133a thereby enhancing cardiac endothelial function and activating the transcription factor AP-2 [137]. In neuroscience, circTulp4 knockout mice revealed that circTulp4 regulates excitatory neurotransmission and modulates sensitivity to aversive stimuli, suggesting its potential as a therapeutic target for anxiety disorders and neurodegenerative diseases [138]. Yao's team successfully administered LNPs encapsulating circSCMH1 *via* the nasal route. CircSCMH1 has been identified as a promoter of angiogenesis through its modulation of the PI3K-Akt and MAPK signaling pathways, and it also facilitates nerve regeneration by influencing extracellular matrix remodeling and axon guidance mechanisms. In a murine model of thrombosis, the treatment demonstrated efficacy in enhancing both sensory and motor function recovery, thereby providing a promising therapeutic approach for ischemic stroke [139]. He et al. [140], starting from clinical issues and the current research status, systematically verified the function of circRMST in patient samples, organoids, PDX and genetically engineered mouse models. They found that circRMST was abnormally highly expressed in neuroendocrine tumors and was highly conserved. It could form a regulatory network by directly binding to transcription factors SOX2 and NKX2-1. On one hand, it maintained the stability of NKX2-1 protein and prevented its degradation through the autophagy-lysosome pathway. On the other hand, it reshaped the genomic binding ability of SOX2, jointly activating the expression of the key neuroendocrine transcription factor ASCL1, thereby driving tumor growth and neuroendocrine transdifferentiation. This broke the traditional perception of circular RNAs as “miRNA sponges” and proposed a new mechanism of “RNA-transcription factor interaction” for regulating gene expression (Fig. 9D).

Additionally, Chen et al. investigated the therapeutic potential of targeting double-stranded circRNAs (ds-cRNAs) to protein kinase R in two mouse models of AD. The findings revealed that the administration of ds-cRNAs into the hippocampus *via* AAV facilitated effective delivery to both neurons and microglia, resulting in a reduction of neuroinflammation and a decrease in β-amyloid plaque accumulation. Furthermore, ds-cRNAs were successfully delivered throughout the entire brain through intravenous injection of AAV-PHP.eB, which can traverse the blood-brain barrier. This approach yielded neuroprotective effects in the AD mouse models and significantly enhanced spatial learning and memory capabilities [141].

Osteoarthritis is a prevalent degenerative joint disorder [142]. While mesenchymal stem cells (MSCs) have been extensively investigated for their potential in osteoarthritis therapy due to their regenerative capabilities and immunomodulatory properties, their effectiveness is constrained by the inherent limitations of the cells' repair mechanisms. A novel lipid nanoparticle system, utilizing the ionizable glycerol lipid TG6A, has been developed to facilitate the efficient delivery of circRNA that encodes fibroblast growth factor 18 (FGF18) to MSC. The modified FGF18-MSCs not only demonstrate a robust capacity to secrete FGF18 protein, thereby promoting the proliferation and matrix synthesis of chondrocytes, but also preserve their immunoregulatory functions. This advancement offers a safe, effective, and translatable approach to cell engineering for the treatment of osteoarthritis [143].

CircRNAs' unique covalently closed structure enhances stability and reduces degradation *in vivo*, making them promising candidates for immune applications. Engineered circRNAs have been developed as protein translation templates, with antigen-encoding circRNAs delivered *via* charge-modified transport proteins. Immunization with these circRNA-based vaccines induced robust innate activation and CD8 T cell responses in mice, highlighting their potential for stimulating effective immune responses [144]. Additionally, circRNA vaccines encapsulated in lipid nanoparticles demonstrated strong antitumor efficacy in murine models by activating both innate and adaptive immunity [145]. CircARSP91 enhance natural killer cell cytotoxicity by upregulating ULBP1, improving immune surveillance and tumor destruction [146] (Fig. 9E). In 2025, Zhu's team developed a small circRNA vaccine (<300 nucleotides) with enhanced thermal and biological stability. Administered via lipid nanoparticles, the vaccine elicited robust T cell responses lasting over six months. Combined with immune checkpoint inhibitors, it significantly suppressed tumor growth, presenting a novel strategy for cancer immunotherapy [147]. Lin et al. [148] developed the first intranasally administered circRNA cancer vaccine using LNPs to encapsulate circRNA. This vaccine induces localized mucosal immune responses. In murine lung cancer models, it elicited robust anti-tumor T cell immunity, precisely eradicating pulmonary tumors while mitigating systemic adverse effects typically associated with intravenously delivered mRNA vaccines. Additionally, the approach can modulate CAR-T cell responses to enhance therapeutic efficacy against tumor cells expressing specific tumor-associated antigens.

CircFAM53B, generated through circularization of exon 2 from the FAM53B gene, is translated in the cytoplasm into a 219-amino acid polypeptide. Its C-terminal 39-amino acid sequence is unique and absent in linear FAM53B mRNAs. Song et al. discovered that the polypeptide translated from circFAM53B can be processed by antigen-presenting cells and loaded onto human leukocyte antigen molecules, eliciting specific cluster of differentiation 4 positive and cluster of differentiation 8 positive T cell responses. Validation in patient-derived immune cell models of breast cancer demonstrated that both circFAM53B and its encoded peptide induce dendritic cell maturation, promote effector T cell differentiation, and significantly enhance expression of cytokines including interferon gamma and granzyme B. In mouse melanoma and breast cancer models, vaccination with dendritic cells pulsed with circFAM53B or its encoded peptide increased tumor-infiltrating cytotoxic T lymphocytes and suppressed tumor growth. Therefore, circFAM53B serves as a prognostic biomarker for breast cancer and melanoma. Tumor-specific circRNAs and their encoded cryptic peptides represent a promising next-generation tumor vaccine strategy, potentially overcoming immunotherapy limitations in patients with low tumor mutational burden [149] (Fig. 9F).

The immunological potential of circRNAs has also been harnessed for vaccine development against viral infections. A 2022 study reported a circRNA vaccine encoding the SARS-CoV-2 spike protein trimeric receptor-binding domain, which elicited neutralizing antibodies and T-cell responses in murine and macaque models, offering protection against SARS-CoV-2 [150]. Wei and colleagues have put forth a patent that outlines a vaccine strategy utilizing circRNAs, which encodes the spike (S) protein of SARS-CoV-2, with the aim of eliciting an immune response to combat COVID-19 [151] (Fig. 9G). Chiu et al. [152] developed monkeypox mixed circular RNA vaccine triggered strong humoral and cellular immunity in mice, and was able to effectively protect immunized mice from monkeypox virus infection. Compared with traditional mRNA, the closed-loop structure of circRNA has high stability, low immunogenicity and low cytotoxicity without modification. Feng et al. [153] developed a single-dose circRNA vaccine prevents Zika virus infection. In this study, compared with the circRNAs encoding monomers or trimers of EDIII, the dimeric EDIII, which is encoded by circRNAs and fused with the human IgG1 Fc fragment (EDIII-Fc), induced better germinal center responses and higher neutralizing antibodies. The circRNAs encoding EDIII-Fc and the non-structural protein NS1 (another protective antigen) of ZIKV were able to prevent lethal ZIKV infection in immunized C57BL/6 mice and adult C57BL/6 mice with knockout of interferon α/β receptors. Importantly, a single-dose optimized circRNAs’ vaccine with improved antigen expression provided effective and durable protection without inducing significant DENV AD in mice (Fig. 9H).

Zhu's team has developed a compact circRNA vaccine with significantly enhanced thermostability and biostability [154]. This circRNA vaccine demonstrates a half-life of 401 days at −20 ​°C, substantially exceeding the 143-day half-life of linear mRNA vaccines and highlighting its superior thermal stability. Delivered *via* LNP carriers, the vaccine induces potent T-cell immune responses persisting for over six months. Monotherapy effectively suppresses tumor growth, while combination therapy with immune checkpoint inhibitors achieves complete regression in 50% of ICB-resistant melanoma cases. This technology overcomes the stability limitations and toxicity challenges inherent in current mRNA vaccines, providing a novel approach for cancer immunotherapy (Fig. 9I).

Wender et al. [155] developed the charge-altering guanidinium-based transporters for RNA delivery. These carriers are designed to degrade guanidinium groups into neutral byproducts, enabling efficient RNA release and translation. The study further revealed a strong correlation between organ tropism and the biophysical properties of nanoparticles, demonstrating that tropism can be modulated by altering the charge ratio in the formulation. GSer-CARTs achieved high-efficiency transfection (>70%) in diverse lung cells and splenic macrophages. Regarding translational potential, delivery of circRNAs encoding ovalbumin protein via GSer-CARTs potently induced cytotoxic antigen-specific T cells and reduced tumor burden.

Li et al. [156], using ionizable lipid U-105 LNP to encapsulate VEGF-A circRNA, a U-LNP/VEGF-A circRNA nanoformulation capable of accelerating the healing of DFU wounds was successfully developed. Compared with linear mRNA, U-LNP/VEGF-A circRNA has better stability and can persistently express active protein both *in vitro* and *in vivo*. U-LNP/VEGF-A circRNA has no cytotoxicity and low immunogenicity. After treatment with a single dose of U-LNP/VEGF-A circRNA for 12 days, the wound of diabetic mice could be almost completely healed, with a significantly better effect than linear VEGF-A mRNA, rhVEGF, and A-LNP/circRNA. Xie et al. [157] developed an innovative therapy based on circRNA, by delivering the mRNA encoding NGF intravitreally to protect retinal ganglion cells. They encapsulated the circRNA expressing NGF in LNPs to form the LNP-circNGF complex, enabling efficient intracellular delivery.

Intervertebral disc degeneration (IVDD) is a chronic degenerative condition where the regenerative potential of nucleus pulposus-resident progenitor cells diminish with aging, rendering them unable to counteract IVDD progression. Jiang's team developed a nucleus pulposus-targeting lipid thymidine nanoparticle (NT-LNP) designed to *in situ* manipulate the regenerative capacity of nucleus pulposus-resident progenitor cells, thereby restoring degenerated nucleus pulposus tissue and alleviating IVDD. Encapsulated within an injectable glycosaminoglycan-based hydrogel for prolonged drug retention, the NT-LNP system concurrently clears inflammatory cytokines MCP-1 and IL-8, improving the degenerative microenvironment. This study represents the first report of an IVDD therapeutic strategy integrating targeted gene delivery with immunomodulatory hydrogels [158]. Meanwhile, Jiang et al. [159] developed a novel chimeric IL-2 signaling receptor (CSR) that enhances the anti-tumor activity of CAR-macrophages against renal cell carcinoma. This study introduces a new approach for solid tumor immunotherapy through innovative CSR design and circRNA technology, combined with a hydrogel-based local delivery system.

### 5.9 Technical bottlenecks and clinical translation challenges

CircRNAs, with their covalently closed structures and diverse biological functions, show great potential in gene regulation, disease mechanisms, and as novel tools for therapy and diagnosis. Compared to linear RNAs' vaccines, circRNAs’ vaccines are more stable, less immunogenic, and enable efficient, sustained protein expression. However, their clinical translation still faces significant challenges.

Synthesis remains a major barrier to clinical application. Although several methods enable efficient *in vitro* production of circRNAs, challenges persist in purification, scalability, and cost. Achieving large-scale, high-purity, and cost-effective manufacturing under GMP conditions is essential, but current technologies are not yet adequate.

The delivery of circRNAs presents significant technical challenges. Their larger size and complex structures, compared to linear mRNAs, hinder efficient encapsulation and stabilization. Conventional vectors such as LNPs, exosomes, viral vectors, and hydrogels require structural optimization or novel designs to ensure effective loading, protection, and controlled intracellular release. Additionally, achieving precise tissue- or cell-specific delivery remains a key hurdle for circRNA-based therapeutics.

Stability and immunogenicity remain important concerns for circRNAs. While their closed structures offer resistance to exonucleases like RNase R, their *in vivo* half-lives and degradation by endonucleases are not fully understood. Moreover, although generally less immunogenic than linear RNAs, synthetic circRNAs can still trigger innate immune responses. For instance, Chen's team showed that certain circRNAs induce immune-related gene expression, depending on their sequence and structure [160].

In clinical translation, circRNAs as a novel therapeutic modality face evolving regulatory landscapes. Agencies like the FDA and EMA are still developing standards for manufacturing, quality control, and clinical study design. Additionally, establishing scalable, GMP-compliant production processes with rigorous quality standards remains a major hurdle.

In summary, the clinical translation of circRNAs still faces several major challenges. These include the need for low cost and high-purity manufacturing processes; strategies to reduce immunogenicity and ensure safety; the development of efficient and targeted delivery systems; a deeper understanding of their biological functions *in vivo*; and overcoming technical and regulatory barriers. Despite these challenges, the intrinsic advantages of circRNAs continue to drive extensive global research and investment, offering strong promise for their future deployment in therapeutic and diagnostic contexts.

As circRNA research transitions from fundamental studies to clinical applications, it introduces a versatile molecular tool with both stability and functional diversity. Future efforts should focus on elucidating its action mechanisms, optimizing delivery platforms, and fostering interdisciplinary collaboration to address existing challenges.

## 6 Conclusion and perspective

This study provides a comprehensive overview of circRNAs, starting from their discovery to an exploration of their biological functions, roles in various diseases, and their potential as therapeutic and diagnostic tools. Particular emphasis has been placed on the synthesis, purification, and delivery systems. as well as advancements in their protein-coding capabilities of circRNAs. While naturally occurring circRNAs have been extensively studied, current technologies now enable precise *in vitro* synthesis, generating synthetic circRNAs with promising applications in research and clinical settings.

The advent of high-throughput screening technologies has significantly expanded the catalog of identified circRNAs, paving the way for their functional characterization and application development. These technologies have not only deepened our understanding of circRNA biology but also highlighted their potential as therapeutic agents and biomarkers. Future research is expected to focus on the directional engineering of circRNAs to optimize their functions for specific disease treatments, leveraging their unique structural and functional properties.

CircRNAs have already demonstrated significant potential as biomarkers for disease diagnosis. For instance, in gastric cancer, the combined use of hsa_circ_0007507 and the serum tumor marker carcinoembryonic antigen provides a more accurate differentiation between patients and healthy individuals compared to carcinoembryonic antigen alone [161]. Similarly, in hepatocellular carcinoma, the integration of alpha-fetoprotein with exo_circ_0006602 improves diagnostic efficacy, as evidenced by an increased area under the curve compared to alpha-fetoprotein alone ^[^ [162]^]^. These findings underscore the diagnostic utility of circRNAs and their potential to complement or even surpass traditional serum tumor markers. In the field of immunotherapy, circRNA-based personalized vaccines exhibit substantial pharmacokinetic advantages over traditional vaccines. These vaccines have shown promise in gene therapy applications, offering a robust platform for stimulating immune responses. However, the research on circRNAs is not without challenges. Key issues include optimizing circRNA synthesis and purification methods, elucidating their underlying mechanisms of action, and ensuring their clinical safety. Despite these hurdles, the field is still in its early stages, and continuous technological advancements are expected to address these challenges.

CircRNAs are poised to become indispensable tools for both research and therapeutic applications. Their stability, diversity, and functional versatility make them attractive candidates for a wide range of applications, from biomarker discovery to drug development and personalized medicine. The translation of circRNA research into clinical reality hinges not only on technological breakthroughs in synthesis and delivery but also on the establishment of standardized validation models. As research progresses, interdisciplinary collaboration and innovative approaches will be essential to fully realize the potential of circRNAs, transforming them into a cornerstone of modern biomedical science.

## CRediT authorship contribution statement

**Xinwei Zhang:** Writing – original draft, Conceptualization. **Hongyan Wu:** Writing – original draft, Validation, Conceptualization. **Xuechuan Hong:** Writing – review & editing, Supervision, Funding acquisition, Conceptualization. **Yuling Xiao:** Writing – review & editing, Project administration, Funding acquisition, Conceptualization. **Xiaodong Zeng:** Writing – review & editing, Project administration, Methodology, Conceptualization.

## Ethics approval

Not applicable.

## Declaration of generative AI in scientific writing

During the preparation of this work the author(s) used ChatGPT in order to assist with language editing and refinement of sentence structure. After using this tool/service, the author(s) reviewed and edited the content as needed and take(s) full responsibility for the content of the publication.

## Funding

The work was supported by the National Key R&D Program of China (2023YFC3605500), 10.13039/501100001809National Natural Science Foundation of China (22477129, 82273796, 82372005, and 82171986), 10.13039/501100010040Taishan Scholar Project of Shandong Province (TSQN202306320), Special Supporting Funds for Leading Talents at or above the Provincial Level in Yantai, 10.13039/501100007129Natural Science Foundation of Shandong Province (ZR2023MB085, ZR2024QH253), Shandong Laboratory Program (SYS202205).

## Data availability

Not applicable.

## Declaration of competing interest

The authors declare that they have no known competing financial interests or personal relationships that could have appeared to influence the work reported in this paper.
